# Understanding how low-income families prioritize elements of health care access for their children via the optimal care model

**DOI:** 10.1186/s12913-014-0585-2

**Published:** 2014-11-19

**Authors:** Heather Angier, Jessica Gregg, Rachel Gold, Courtney Crawford, Melinda Davis, Jennifer E DeVoe

**Affiliations:** Family Medicine, Oregon Health & Science University, Portland, OR USA; Internal Medicine, Oregon Health & Science University, Portland, OR USA; Center for Health Research, Kaiser Permanente Northwest, Portland, OR USA; Research, OCHIN, Inc., Portland, OR USA

**Keywords:** Health insurance, Access to care, Children’s health, Qualitative research

## Abstract

**Background:**

Insurance coverage alone does not guarantee access to needed health care. Few studies have explored what “access” means to low-income families, nor have they examined how elements of access are prioritized when availability, affordability, and acceptability are not all achievable. Therefore, we explored low-income parents’ perspectives on accessing health care.

**Methods:**

In-depth interviews with a purposeful sample of 29 Oregon parents who responded to a previously administered statewide survey about health insurance. Transcribed interviews were analyzed by a multidisciplinary team using a standard iterative process.

**Results:**

Parents highlighted affordability and limited availability as barriers to care; a continuous relationship with a health care provider helped them overcome these barriers. Parents also described the difficult decisions they made between affordability and acceptability in order to get the best care they could for their children. We present a new conceptual model to explain these experiences accessing care with health insurance: the Optimal Care Model. The model shows a transition from *optimal care* to a *breaking point* where affordability becomes the driving factor, but the care is perceived as unacceptable because it is with an unknown provider.

**Conclusions:**

Even when covered by health insurance, low-income parents face barriers to accessing health care for their children. As the Affordable Care Act and other policies increase coverage options across the United States, many Americans may experience similar barriers and facilitators to health care access. The Optimal Care Model provides a useful construct for better understanding experiences that may be encountered when the newly insured attempt to access available, acceptable, and affordable health care services.

## Background

The Children’s Health Insurance Program (CHIP) has contributed to an increase in the number of US children with coverage [1–3]. The Affordable Care Act (ACA) will increase coverage options for many adults through Medicaid expansions and health insurance exchanges [4]. However, insurance coverage alone does not guarantee access to needed health care [5–8]. In addition to coverage barriers, previous research has demonstrated the presence of both financial barriers and structural facilitators (e.g., clinic or provider takes your insurance, you have a usual source of care) to optimal receipt of health care [5,6,9–12]. For example, high deductibles and co-pays prevent many privately insured low- and middle-income families from getting necessary care [5], and medical debt, despite insurance coverage, predicts reduced access to care [10]. Additionally, when children have both health insurance and a usual source of care, they are less likely to have unmet healthcare needs [13] and are more likely to be appropriately immunized [14].

Conceptual models that describe factors contributing to health care access include availability, affordability, and acceptability of care [15–19]. Availability is the adequacy of supply of providers, clinic sites, and programs. Affordability is the relationship between prices and a patient’s ability to pay. Acceptability refers to patient attitudes about provider and/or clinic site attributes [18,19]. Anderson and Aday (1974) created a framework that focused on the relationship between the characteristics of the health care system and those of the population at risk, specifically the enabling resources of the system and the predisposing factors of the population [15]. These early conceptual models of health care access also acknowledge that to be accessible, health care must be appropriate to the individual needs of the person and family seeking care [15–19].

Despite recognition of the importance of the patient perspective in understanding access to care, few studies have explored in-depth the meaning of “access” to low-income families, nor have they examined how these families prioritize access elements, including availability, affordability, and acceptability, when not all are achievable. Therefore, we explored low-income parent’s experiences obtaining health care for their children to better understand the barriers to and facilitators of access and how families prioritize important access elements.

## Methods

### Participants

We selected a purposeful sample of low-income households from a statewide cohort of parents who participated in the Oregon Children’s Access to Healthcare Survey. The specific sampling procedures and findings of that survey are reported elsewhere [20,21]. Briefly, families eligible for food assistance were selected to participate in the survey, as the Oregon Health Plan [(OHP), which includes Oregon’s Medicaid program and other similar public insurance programs] utilizes the same eligibility criteria. From the original survey of 2,681 households, we selected a subset of parents based on location (one hour drive of our academic institution), language (spoke English or Spanish), and insurance status of their children (public or private insurance). In June 2010, we mailed an informational letter to all eligible parents (N = 360) to invite them to participate in an interview. We initiated follow-up phone calls two weeks later to set up interviews with everyone who was sent an informational letter. We stopped data collection after 25 English and four Spanish interviews when saturation, the point at which findings repeat or recur, was achieved [22,23].

### Data collection

Between July 2010 and January 2011, three trained research assistants conducted interviews using a semi-structured interview guide. The guide was developed by the multidisciplinary team and informed by a review of health care access conceptual models [15,17–19]. Open-ended questions were designed to explore parents’ perceptions of the barriers to and facilitators of children’s access to health care services, as well as information on how they prioritized important access elements. Specific questions about availability, affordability, and acceptability of health care were included. Other questions dealt with general thoughts on health care and current reform efforts. Interviewers asked questions in the same sequence and used inductive probing on responses requiring additional information.

Consent was obtained prior to each interview. Interviews lasted 60–90 minutes, were digitally recorded, transcribed verbatim and re-read by the interviewer for accuracy. Spanish interviews were conducted by a Spanish-speaking interviewer, transcribed into English by a medical transcriptionist certified in Spanish-English translation and re-read by the interviewer for accuracy. Participants received a $25 gift card for participating.

### Data analysis

We conducted descriptive analysis of participant demographic information from the original survey responses using SPSS software, version 18.0 (Chicago, SPSS, Inc.). MAXQDA software, version 10.0 (Udo Kuckartz, Berlin) was used for qualitative data management and analysis. Our multidisciplinary analysis team included a physician researcher-anthropologist with extensive qualitative analysis expertise, a Ph.D. health services researcher skilled in quantitative and qualitative research, and a research associate with qualitative research experience. We analyzed the data using a modified version of focused coding and grounded theory method, both of which are reproducible and scientifically rigorous [24,25]. After each team member independently read all transcripts, we met to discuss conceptual codes using an iterative process and created an initial thematic codebook [26]. We held a series of meetings to allow additional codes to surface and revised the codebook accordingly. Two team members independently coded five transcripts then met to resolve discrepancies using a consensus approach and finalize the codebook. The remaining transcripts were coded by one individual and reviewed by the second team member for agreement. These processes allowed us to examine the data and then reflect on it until definitive patterns emerged and saturation achieved [22]. We then used a deductive approach to identify emergent themes that related existing conceptual models. The study protocol was reviewed and approved by the Oregon Health & Science University Institutional Review Board (IRB#00001717).

## Results

### Participant demographics

As summarized in Table 1, over 90% of the participants had incomes less than 133% of the federal poverty level. Of the parents interviewed, 86% spoke English as their primary language and 14% spoke Spanish; 25% lived in rural areas near Portland, Oregon. Additionally, 62% of the parents reported their children to be White, non-Hispanic; 21% Hispanic, any race; and 17% non-White, non-Hispanic. At the time of the interview, 18 participating parents reported their children had public health insurance coverage.

**Table 1:** **Demographics of interviewed participants**

**Child race/ethnicity**	**%**
White, non-Hispanic	62.1
Hispanic, any race	20.7
Non-white, non-Hispanic	17.2
**Federal Poverty Level (FPL)**	
Zero	13.9
1-50%	37.9
51-100%	20.7
101-133%	20.7
>133%	6.9
**Language**	
English	86.2
Spanish	13.8
**Location of residence**	
Urban	75.9
Rural	24.1

N = 29.

Source: Oregon Children’s Access to Healthcare Survey.

### Emergent themes

Three main themes emerged in the data: 1) affordability and availability were the most pronounced barriers shaping access to care; 2) a continuous relationship with a health care provider or clinic site was the most important facilitator in overcoming access barriers, including challenges associated with affordability and availability; 3) parents wanted access to optimal care, which they described as including affordability, availability, and acceptability. However, many parents were faced with making difficult decisions between these ideals. In most cases, parents tried to maintain a relationship with a provider, and preserving this relationship helped them overcome other barriers they faced accessing health care. However, many described a time when they could no longer maintain a continuous relationship with a provider due to affordability. Each theme is described below in greater detail with illustrative quotations, and a conceptual model of theme 3 (the Optimal Care Model) is presented.

#### Theme 1: Barriers in access to care: affordability and availability

Even with health insurance coverage, low-income parents identified difficulties affording care for their children due to the high cost of deductibles, co-payments, and/or medications.

*You don’t realize how expensive it is until you’re paying at every doctor visit. We were thankful we did have some kind of government health plan to help.*

*I don’t always have the money to pay the deductible. [My kids] have great insurance, but [the deductible] makes me think twice about taking them to the doctor…*

*Besides that [health insurance premium] I have…a $25 co-pay. So, there are some times when I have to wait until I get paid to make an appointment [for my children]. So, if… we need to see the doctor, I schedule the appointment around the time when I get paid so we can pay the co-pay and it is kind of frustrating sometimes.”*

*“Medicine is very expensive. I have to go to the doctor and pay my co-pay but don’t have money to pay for the medications [my children need].*

In addition to cost, public coverage was also identified as a potential barrier to attaining care,

*[When obtaining care] the only time I felt [treated differently] is when I have tried to find [my children] a clinic or doctor to go to. Then I hear, ‘we don’t take OHP’ or ‘we’re no longer taking OHP patients.’*

One described the experience of limited availability of providers who accepted public health insurance as *“a cattle call”* in which all the individuals with public insurance rushed to the few providers who would accept it. Additional participants stated,

*It was just hard to get people to take that [public] insurance.*

*Some doctors don’t take OHP after a while because they have too many patients on it.*

*They told me: ‘you can’t even walk in this facility because Medicaid is your co-pay.*

*I’ve had them not even give me a referral when they see we’re on OHP.*

#### Theme 2: Facilitating factor to accessing care: a continuous relationship with a provider or clinic

Parents reported a continuous relationship with a primary care provider or clinic site helped them overcome barriers to accessing health care for their children. One participant recounted that her primary care provider helped her get insurance approval for physical therapy needed by her daughter:

*Sometimes if a provider calls…you’re going to get a different result. Sure enough, she got us to talk to the right people who didn’t totally blow us off. They told me if [my daughter] had not been an existing patient, they wouldn't have seen [her]. Luckily I had formed a relationship with [the provider when] she had insurance. If she hadn’t…I would have had nowhere to go other than a random emergency room.*

Many parents told stories of incidents where they felt they were *only* able to obtain care for their children on OHP because of existing relationships.

*I knew they didn’t usually take OHP. [I said to him] ‘But I’ve already seen you. You know about my complications. Can you please take my OHP?’ He said he would.*

*It was about that time [daughter’s name] broke her ankle. I think we’re fortunate in that we had an established physician. We went to him…and they accepted the plan. There wasn’t any problem with that.*

Having a trusted provider or clinic site was instrumental in helping many low-income parents overcome affordability and availability barriers. Parents were able to get their children seen when needed because they had an established relationship regardless of their insurance type or how long it would take them to pay the bill.

#### Theme 3: The relationship between accessible, perceived acceptable, and affordable care

Optimal care was important to the low-income parents we interviewed. Specifically, they described optimal care as affordable, available health services for their children with a provider with whom they felt comfortable and had an established, trusted relationship.

*The doctor we were already seeing accepts OHP. It was really easy that way. I can just imagine not having a provider that would accept it…We got really lucky.”*

*When I’ve been on it [OHP], it’s been fine. I can get what I need, the services I need are taken care of…Once I have been on it it’s been fine.*

Many parents, however, described reaching a point at which they were forced to make difficult choices between a continuous relationship with a provider, affordability, and availability – and that such choices resulted in tradeoffs that yielded what was perceived as unacceptable care. Once faced with this point, parents engaged in a process of evaluating and re-evaluating how to prioritize limited resources; for example, prioritizing among a trusted relationship versus affordability versus availability.

*The real issue is: ‘who is going to take the best care of your child and can you afford it?’ It goes back and forth, that is where we are today. It hasn’t always been that way. …The reality is, if you do it on a month by month basis and you take worst case scenarios and put pencil to paper, you say, oh, this works better for me.*

Most parents prioritized a continuous relationship with a primary care provider or clinic, if possible. Parents often felt frustrated when they were reassigned providers or designated with a new clinician because of a change in their insurance status.

*That part drives me nuts - you can find a really good doctor but as soon as your insurance changes, you’re out of luck. You have to switch.*

The two quotes below exemplify the trade-offs that parents must make when accessible, perceived acceptable, and affordable care are not all possible:

*When I got the…cards in the mail, they had automatically generated a provider on it based on my zip code… I knew nothing about it, nothing about the provider and I was a little thrown by it. Who decided…I wanted to keep the kids where they had always been. You can tell me this is close to you. I will drive farther if I feel I’m going to get good service or convenient hours.*

*[Even though insurance would not pay] I’m not changing her doctors, that’s not fair to her or me just because I moved. I had to change every specialist she’s ever seen? That’s stupid. They’re doing really well with her right now. They know her whole health history. To start that all over?*

Parents also described a point at which they were no longer able to make choices involving prioritization. At this point, parents described having to “settle for” affordable care that they perceived unacceptable. This was the point when they were forced to abandon all other factors for affordability.

*The deductible is $300 per person and the co-pay, they pay 90%. I don’t remember what the premium is but I know there is no deductible, which is amazing, which is why I switched. It’s only the $10 co-pay. Financially, that is more reasonable and I feel that is going to be better off for us, but I had to do a lot of thinking about that… I am substituting quality, I’m giving up my daughter’s doctor, who I love--who has been there for both of [my children] since birth--to go to a complete unknown that I haven’t heard the best things about.*

### A new conceptual model: the optimal care model

**Figure 1:**
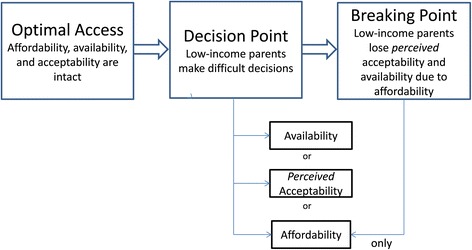
**The optimal care model.**

Findings from our interviews revealed a new conceptual model of access to care. As described in Figure 1, this model starts with *optimal access* where parents are able to obtain care without choosing among access factors including availability, affordability, and acceptability. Next, we describe a “*decision point,”* the critical juncture at which parents are pushed into making difficult choices between access elements to obtain care. The decision point is where parents experience affordability and availability barriers and may turn to trusted providers to help them overcome these barriers. Many parents are willing to drive further or pay more to maintain a continuity relationship that could help them mitigate barriers to access. Last, we present a “*breaking point.”* At this point, health care services are no longer affordable and parents are forced to sacrifice all other access factors. Parents described feeling hopeless when they reached this point because they believe they must sacrifice optimal care for affordability.

## Discussion

This study found affordability and lack of availability to be the most commonly reported barriers to health care access for low-income parents. They described many instances where, despite their child having health insurance, they were unable to receive necessary health care services. This happened most often because they could not find a provider to accept public health insurance or because they could not afford the co-payment due at the time of service. Results of these interviews confirm those of previous studies that identified the lack of affordable and available care as major access barriers [27–29]. We found the most important factor to help parents overcome these access barriers was a continuous relationship with a health care provider or clinic site. Participants in this study described how health care providers helped them overcome access barriers to receive needed care. Previous research has also recognized the importance of established relationships with trusted health care providers to mitigate barriers [30–35]. We organized the concepts described to us into a new conceptual model called the Optimal Care Model (Figure 1). We believe this adds a perspective critical to understanding what many low-income parents experience when trying to obtain health care for their children.

### Policy implications

The ACA has introduced health insurance exchanges and many states have expanded Medicaid coverage [4]. These policy changes will increase coverage opportunities for more Americans, yet many of the barriers to receiving health care may persist and families may face difficult decisions in order to receive optimal care. The Optimal Care Model provides a useful construct for better understanding families’ experiences in gaining access to health care. Health insurance coverage alone did not guarantee optimal access to health care and parents described times when they were not able to obtain “optimal” health care for their children; they talked about how they prioritized factors when they could not achieve such care. The ACA is taking important steps to ensure health care access, especially for low-income households, including expanding health insurance coverage, increasing the primary care workforce, and increasing Medicaid reimbursement [4,36–38]. Additional policies that caution against the movement of patients from one provider to another could further increase optimal access.

### Limitations

Our study has several limitations. We conducted the interviews in one region of one state with a cohort of parents who responded to a prior survey; therefore, the experiences described may reflect barriers encountered only by this population. The sample included a demographically diverse group (parents had similar demographics as their children) with interviews conducted in both English and Spanish, but Oregon has fewer ethnic and racial minorities than many other states. Interviews were conducted by three interviewers (one Spanish speaker), which could potentially contribute to response variability. We contacted participants several years after the initial survey and many potential participants had moved, leaving no forwarding addresses or telephone numbers, which may have biased our sample towards families who move less often. We tried to address these limitations by attempting to locate current addresses and telephone numbers for eligible participants, utilizing a standardized, semi-structured interview guide, conducting interviews until we found saturation of themes, and having multiple individuals participate in the analysis. Despite these potential limitations, this study provides a compelling description of the experiences low-income Oregon parents face in accessing health care for their children; which may also apply in other states.

## Conclusion

Even when covered by health insurance, low-income parents face barriers to accessing health care for their children. Affordability and availability are common barriers that can be mitigated by having a continuous relationship with a health care provider or a clinic. We organized the themes described to us by participants into a new conceptual model, the Optimal Care Model, which demonstrates the difficult decisions that some families face when accessing health care for their children. This model also provides insights about what Americans who gain coverage through ACA insurance expansions might face in the near future.

## Abbreviations

(CHIP): Children’s Health Insurance Program
(ACA): The Affordable Care Act
(OHP): Oregon Health Plan

## References

[1] **Children's Health Insurance Program Reauthorization Act of 2009 (CHIPRA).** http://kaiserfamilyfoundation.files.wordpress.com/2013/01/7863.pdf.

[2] **Children's Health Insurance Program Reauthorization Act of 2009.** http://www.gpo.gov/fdsys/pkg/PLAW-111publ3/html/PLAW-111publ3.htm.

[3] Cuttler L, Kenney G (2007). State children's health insurance program and pediatrics. Arch Pediatr Adolesc Med.

[4] **Compilation of Patient Protection and Affordable Care Act.** http://housedocs.house.gov/energycommerce/ppacacon.pdf.

[5] DeVoe JE, Baez A, Angier H, Krois L, Edlund C, Carney PA (2007). Insurance + access not equal to health care: typology of barriers to health care access for low-income families. Ann Fam Med.

[6] DeVoe JE, Graham AS, Angier H, Baez A, Krois L (2008). Obtaining healthcare services for low-income children: a hierarchy of needs. J Healthcare Poor Underserved.

[7] Newacheck P, Hughes D, Stoddard J (1996). Children's access to primary care: differences by race, income, and insurance status. Pediatrics.

[8] Newacheck PW, Hughes DC, Hung Y-Y, Wong S, Stoddard JJ (2000). The unmet health needs of America's children. Pediatrics.

[9] Sobo EJ, Seid M, Reyes Gelhard L (2006). Parent-identified barriers to pediatric health care: a process-oriented model. Health Serv Res.

[10] Herman PM, Rissi JJ, Walsh ME (2011). Health insurance status, medical debt, and their impact on access to care in Arizona. Am J Public Health.

[11] Kullgren JT, McLaughlin CG (2010). Beyond affordability: the impact of nonfinancial barriers on access for uninsured adults in three diverse communities. J Community Health.

[12] Kullgren JT, McLaughlin CG, Mitra N, Armstrong K (2012). Nonfinancial barriers and access to care for U.S. adults. Health Serv Res.

[13] DeVoe JE, Tillotson C, Lesko SE, Wallace L, Angier H (2011). The case for synergy between a usual source of care and health insurance coverage. J Gen Intern Med.

[14] Dombkowski KJ, Lantz PM, Freed GL (2004). Role of health insurance and a usual source of medical care in age-appropriate vaccination. Am J Public Health.

[15] Aday LA, Andersen R (1974). A framework for the study of access to medical care. Health Serv Res.

[16] Carillo JE, Carillo VA, Perez HR, Salas-Lopez D, Natale-Pereira A, Byron AT (2011). Defining and targeting health care access barriers. J Health Care Poor Underserved.

[17] Andersen R (1995). Revisiting the behavioral model and access to medical care: does it matter?. J Health Soc Behav.

[18] Penchansky R, Thomas WJ (1981). The concept of access definition and relationship to consumer satisfaction. Med Care.

[19] Gulliford M, Figueroa-Munoz J, Morgan M, Hughes D, Gibson B, Beech R, Hudson M (2002). What does 'access to health care' mean?. J Health Serv Res Policy.

[20] DeVoe JE, Lisa K, Tina E, Jeanene S, Nichole EC (2008). Uninsurance among children whose parents Are losing Medicaid coverage: results from a statewide survey of Oregon families. Health Serv Res.

[21] DeVoe JE, Krois L, Edlund C, Smith J, Carlson NE (2008). Uninsured but eligible children: Are their parents insured? Recent findings from Oregon. Med Care.

[22] Guest G, Bunce A, Johnson L (2006). How many interviews are enough? An experiment with data saturation and variability. Field Methods.

[23] Crestwell JW (2007). Qualitative Inquiry & Research Design: Choosing Among Five Approaches - 2nd Edition.

[24] Miles MB, Huberman AM (1994). Qualitative Data Analysis.

[25] Strauss A, Corbin J (1990). Basics of Qualitative Research: Grounded Theory.

[26] MacQueen K, McLellan E, Kay K, Milstein B (1998). Codebook development for team-based qualitative analysis. Cultural Anthropol Meth J.

[27] Bashshur RL, Homan RKMA, Smithe DG (1994). Beyond the uninsured: problems in access to care. Med Care.

[28] Bisgaier J, Rhodes KV (2011). Auditing access to specialty care for children with public insurance. N Engl J Med.

[29] Bisgaier J, Polsky D, Rhodes KV (2012). Academic medical centers and equity in specialty care access for children. Arch Pediatr Adolesc Med.

[30] DeVoe JE, Petering R, Krois L (2008). A usual source of care: supplement or substitute for health insurance among low-income children?. Med Care.

[31] DeVoe JE, Tillotson CJ, Wallace LS, Lesko SE, Pandhi N (2012). Is health insurance enough? A usual source of care may be more important to ensure a child receives preventive health counseling. Matern Child Health J.

[32] Sox CM, Swartz K, Burstin HR, Brennan TA (1998). Insurance or a regular physician: which is the most powerful predictor of health care?. Am J Public Health.

[33] Eisert SL, Durfee MJ, Welsh A, Moore SL, Mehler PS, Gabow PA (2009). Changes in insurance status and access to care in an integrated safety net healthcare system. J Community Health.

[34] Smith LH, Holloman C (2011). Comparing child health, access to care, and utilization of health services between Ohio Appalachia's River and non-river bordering counties. J Community Health.

[35] O'Malley ASF CB (1996). Continuity of care and delivery of ambulatory services to children in community health clinics. J Community Health.

[36] Ku L, Jones K, Shin P, Bruen B, Hayes K (2011). The states' next challenge–securing primary care for expanded Medicaid populations. N Engl J Med.

[37] Zweifler J, Prado K, Metchnikoff C (2011). Creating an effective and efficient publicly sponsored health care delivery system. J Health Care Poor Underserved.

[38] Shaffer ER (2013). The affordable care act: the value of systemic disruption. Am J Public Health.

